# Supplementary material for: Coupling remote sensing and eDNA to monitor environmental impact: A pilot to quantify the environmental benefits of sustainable agriculture in the Brazilian Amazon
Source: PLoS One. 2024 Feb 14;19(2):e0289437. doi: 10.1371/journal.pone.0289437 (PMC10866516; doi:10.1371/journal.pone.0289437)
Supplement: S1 Table — Sample size per class for validation efforts. The change maps (disturbance map and regeneration map) were combined into one map and the same sample dataset was used for accuracy assessment. (DOCX) [file pone.0289437.s001.docx]

# SAMPLE SIZES FOR CHANGE MAP VALIDATION

The sample size per class for both validation datasets is found in Table S1.

*Table S1: Sample size per class for validation efforts. The change maps (disturbance map and regeneration map) were combined into one map and the same sample dataset was used for accuracy assessment.*

| **Change maps (disturbance and regeneration)** | |
| --- | --- |
| **Class** | **Validation sample (n)** |
| Degradation | 100 |
| Stable non-forest | 100 |
| Stable forest | 100 |
| Deforestation | 100 |
| Regrowth | 100 |
| Different events | 100 |
| **Total** | 600 |
