# Supplementary material for: Coupling remote sensing and eDNA to monitor environmental impact: A pilot to quantify the environmental benefits of sustainable agriculture in the Brazilian Amazon
Source: PLoS One. 2024 Feb 14;19(2):e0289437. doi: 10.1371/journal.pone.0289437 (PMC10866516; doi:10.1371/journal.pone.0289437)
Supplement: S2 Table — Morphological Spatial Pattern Analysis of forest change dynamics from 2001 to 2019 in the study area. (DOCX) [file pone.0289437.s002.docx]

# DETAILED MSPA RESULTS

*Table S2. Morphological Spatial Pattern Analysis of forest change dynamics from 2001 to 2019 in the study area.*

|  | Area (ha) within shaded-grown farm properties |
| --- | --- |
| Patch forest | 114 |
| Outer edge (stable) | 1,000 |
| Inner edge (stable) | 82 |
| Core forest | 93 |
| Secondary degradation | 1,189 |
| Secondary deforestation | 3,154 |
| Primary degradation | 737 |
| Primary deforestation | 834 |
