# Supplementary material for: Coupling remote sensing and eDNA to monitor environmental impact: A pilot to quantify the environmental benefits of sustainable agriculture in the Brazilian Amazon
Source: PLoS One. 2024 Feb 14;19(2):e0289437. doi: 10.1371/journal.pone.0289437 (PMC10866516; doi:10.1371/journal.pone.0289437)
Supplement: S3 Table — Planned sampling and plots returning usable data after filtering. (DOCX) [file pone.0289437.s003.docx]

# BIODIVERSITY RESULTS BY SITE

*Table S3: Planned sampling and plots returning usable data after filtering.*

| Site | Planned plots | Sampled plots | Plots returning data | Plots with data after filtering (2g verts) | Plots with data after filtering (2g arthropods) | Data collected on (date) |
| --- | --- | --- | --- | --- | --- | --- |
| Cocoa 5 | 4 | 4 | 4 | 2 | 4 | June 30, 2021 |
| Cocoa 2 | 4 | 4 | 3 | 0 | 4 | July 5, 2021 |
| Cocoa 4 | 4 | 4 | 4 | 0 | 4 | June 24, 2021 |
| Cocoa 1 | 3 | 3 | 3 | 0 | 3 |  |
| Cocoa 3 | 4 | 3 | 3 | 2 | 3 | June 29, 2021 |
| Pasture 3 | 4 | 3 | 2 (vert) 3 (arthr.) | 2 | 3 | July 8, 2021 |
| Pasture 2 | 3 | 3 | 3 | 2 | 3 | June 23, 2021 |
| Pasture 5 | 4 | 4 | 3 | 3 | 4 | June 23, 2021 |
| Pasture 1 | 4 | 4 | 4 | 1 | 4 (2 over 2k reads) | July 6, 2021 (palms + growth) |
| Pasture 4 | 4 | 3 | 3 | 3 | 3 | July 7, 2021 |
| Forest 1 | 4 | 3 | 3 | 3 | 3 | June 25, 2021 |
