# Supplementary material for: Coupling remote sensing and eDNA to monitor environmental impact: A pilot to quantify the environmental benefits of sustainable agriculture in the Brazilian Amazon
Source: PLoS One. 2024 Feb 14;19(2):e0289437. doi: 10.1371/journal.pone.0289437 (PMC10866516; doi:10.1371/journal.pone.0289437)
Supplement: S1 File — A detailed description of all MPSA definitions. (DOCX) [file pone.0289437.s004.docx]

# MSPA DEFINITIONS

- Core forest: Core pixels are defined as those foreground pixels whose distance to the background is greater than a given size-parameter and are stable over time.
- Patch forest: Patch forest pixels are defined as those connected components of foreground pixels that do not contain any core forest pixels and are stable over time.
- Boundary pixels (inner and outer edge): Boundaries are defined as those foreground pixels that separate the core pixels from the background pixels and are stable over time.
  - Inner edge: Inner edge pixels of a given connected component are defined as its boundary pixels that are within a distance to a hole of this connected component where a hole is defined as a connected component of the background that does not contain any pixel of the border of the image and are stable over time.
  - Outer edge: Outer edge pixels of the connected component are obtained by subtracting its inner edge pixels from its boundary pixels.
- Secondary degradation: Foreground pixels that transitioned from inner edge to outer edge, or from inner/outer edges to patch forest.
- Secondary deforestation: Foreground pixels that transitioned from patch or inner/outer edge to non-forest (background pixel)
- Primary degradation: Foreground pixel that transitioned from core forest to patch forest or inner/outer edge.
- Primary deforestation: Foreground pixels that transitioned from core forest to non-forest (background pixel).

Definitions adapted from:

- Soille P. and Vogt P. (2022). [Morphological spatial pattern analysis: open source release](https://doi.org/10.5194/isprs-archives-XLVIII-4-W1-2022-427-2022). The International Archives of the Photogrammetry, Remote Sensing and Spatial Information Sciences, Volume XLVIII-4/W1-2022 Free
- Open Source Software for Geospatial (FOSS4G) 2022 – Academic Track, 22–28 August 2022, Florence, Italy. pp. 427-433, doi:doi.org/10.5194/isprs-archives-XLVIII-4-W1-2022-427-2022
