# Supplementary material for: Coupling remote sensing and eDNA to monitor environmental impact: A pilot to quantify the environmental benefits of sustainable agriculture in the Brazilian Amazon
Source: PLoS One. 2024 Feb 14;19(2):e0289437. doi: 10.1371/journal.pone.0289437 (PMC10866516; doi:10.1371/journal.pone.0289437)

**Protocol for Collecting Environmental DNA Samples From Soils**

Pilot study - TerraBio: a remote sensing and biodiversity monitoring tool for business performance evaluation and decision-making

Naiara Sales & Allan McDevitt

University of Salford

April 2021


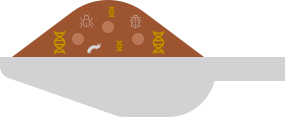


Brief overview

This pilot study aims to collect environmental DNA from soil samples and investigate its potential in assessing the invertebrate biodiversity present in distinct environmental settings (secondary forest patches, shaded cocoa plots and pasture areas).

Ultimately, we aim to test for variation in entomofauna assemblages that are associated with different land uses in the Amazon.

Sampling tips:

The most important step to ensure the accuracy of eDNA results is to avoid contamination of the field sample. Therefore, care should be taken when collecting the samples, especially when considering different locations/systems.

- Be careful with gloves and all other supplies. Do not leave them unprotected and do not keep them loose in a backpack. Keep everything clean and organised in plastic bags.

- Do not touch anything other than decontaminated materials while sampling. If your gloves touch anything that you’re not certain is clean, replace them with clean gloves.

- Collect samples where the soil has not yet been stepped on to avoid cross-contamination between sites.

How to decontaminate tools:

Decontaminate tools between each use by placing them in 50% bleach for at least 1 minute. **Rinse well** with distilled water to remove all bleach residues (remaining bleach residues may degrade the DNA and hamper species detection).

If using disposable tools: use new ones for each sample, discarding right after use (in the black plastic bag - to avoid contaminations).

Sampling Kit

- Non-powder disposable gloves (e.g., nitrile gloves). To be discarded after each sample collection to avoid contamination.
- Permanent markers
- Sampling tools (e.g., [scoops](https://www.prolab.com.br/produtos/materiais-de-plastico/espatula-de-plastico/espatula-em-polipropileno-concha/), trowel, or centrifuge tubes), either stainless steel or disposable plastic (preferred option).

*If using steel tools: Please follow decontamination procedures as described above. Example:* [*50 mL tubes*](https://www.casalab.com.br/produtos/92/13909_) *with 30 mL of 50% bleach solution (15 ml household bleach and 15 mL water) and water to decontaminate tools between sites (distilled, deionized or municipally treated tap water).*

- Silica bags
- Whirl-pak sampling bags (e.g., [Merck](https://www.sigmaaldrich.com/catalog/substance/whirlpaksamplebag1234598765?lang=pt&region=PT), [CasaLab](https://www.casalab.com.br/produtos/92/12241_))
- Storage boxes (plastic containers to store and ship samples)
- Portable digital scale (or another viable solution to measure/weight soil samples)
- Black bag for used tools (i.e., to discard all used/contaminated disposable tools during the field sampling).


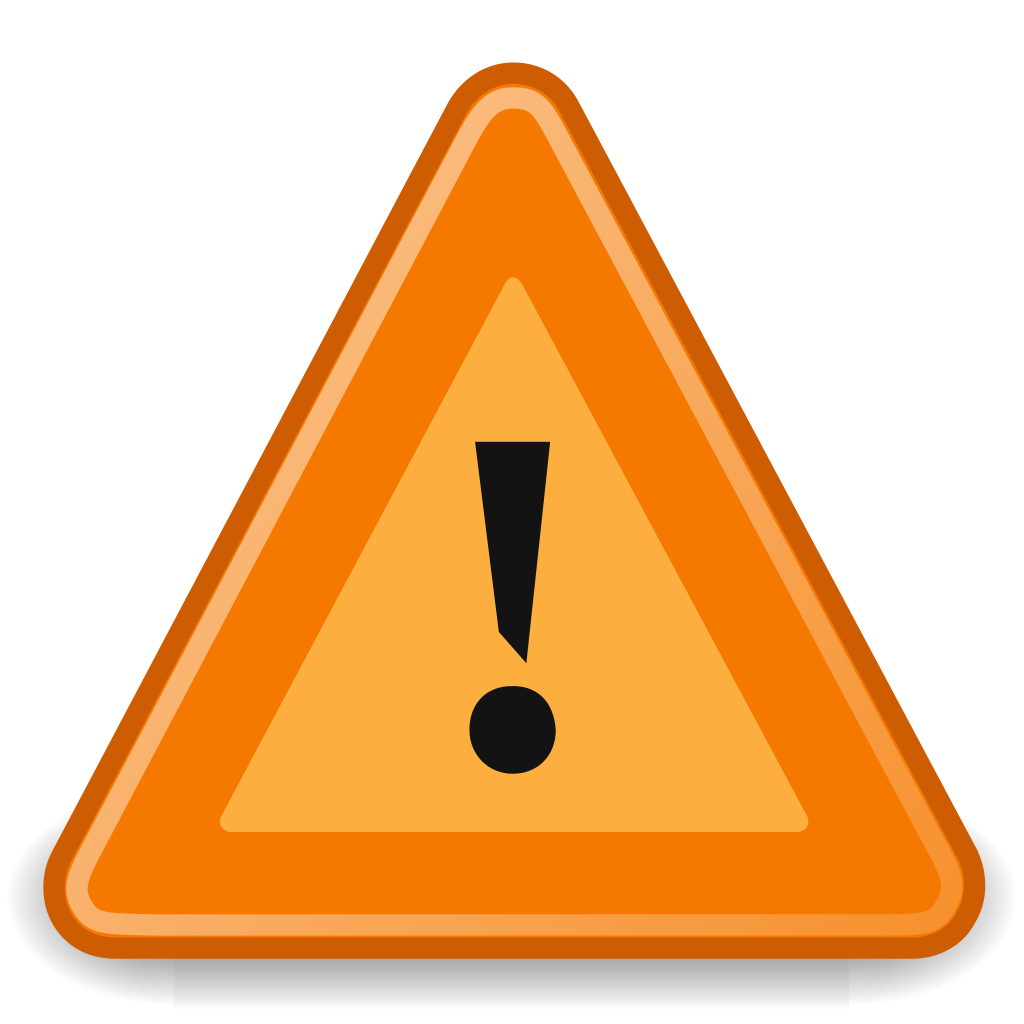


Keep used/contaminated items completely separated from the new/unused items to avoid cross-contamination.


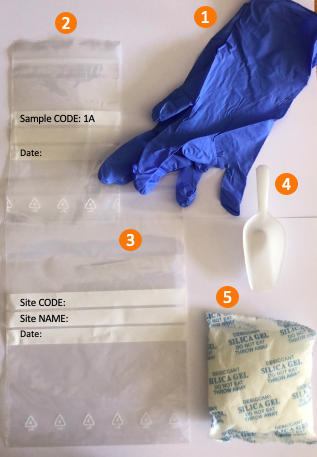


Figure 1: Example of materials required for soil sampling.

1. Nitrile gloves (disposable)
2. Small sampling bag (to store individual samples)
3. Large sampling bag (to store replicates/multiple samples from the **same collection site**).
4. Soil collection disposable tool
5. Silica bag (smaller silica bags are recommended - to be included in the small sampling bags and preserve the soil samples)


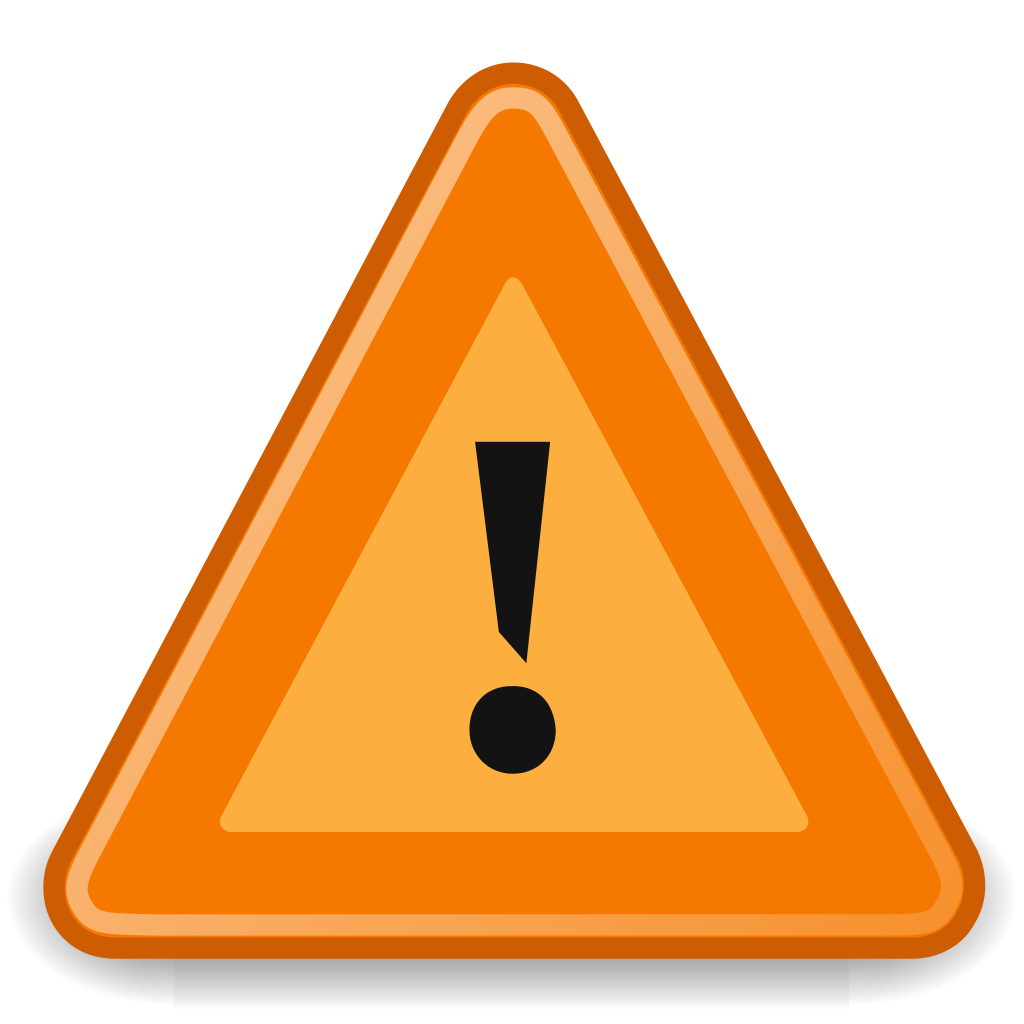


All relevant information should be included in a file matching the sampling codes.

Environmental DNA is not homogeneously distributed within terrestrial systems, therefore within-site sample location can be important for detection. In this context, for the same site plot, four replicates will be collected as shown in Fig.2.

1. Before starting the fieldwork, prepare sampling kits for each site (including labelled sampling bags). Each sample should have a unique code. Sites can be labelled using collection location names and sequential numbers. Locations/replicates within each site can be labelled using names and letters.

*Example: Site 1, replicate 1 → 1A*

Replicates from the same site can be placed together in a single bag. But samples from different collection sites should be kept separately.

*Example: Bag labelled 'Site 1' (containing samples 1A, 1B, 1C, 1D)*

*Bag labelled 'Site 2' (containing samples 2A, 2B, 2C, 2D)*

1. After arriving at the sampling site, wear new gloves and grab the pre-labelled Whirl-Paks sampling bags and collection tools.
2. Using the sampling tool (disposable or previously decontaminated) collect ~30g of soil from the top layer (0-10 cm depth). Avoid collecting leaf litter and superficial layers.
3. Place the collected sample in the labelled bag and seal it.
4. After finishing the soil sampling, store the plastic bag in a separate container (or larger plastic bag), and discard the used gloves and disposable tools.
5. If using steel tools, decontamination should be conducted before starting the new sampling when arriving at the sampling site. Decontaminate tools in 50% bleach for at least 1 minute. Rinse well with water.

Environmental DNA samples are usually stable in silica desiccant beads for several weeks. However, it is important to keep them away from water, heat, and sunlight. Sample bags should be kept in a freezer until shipping to the University of Salford.


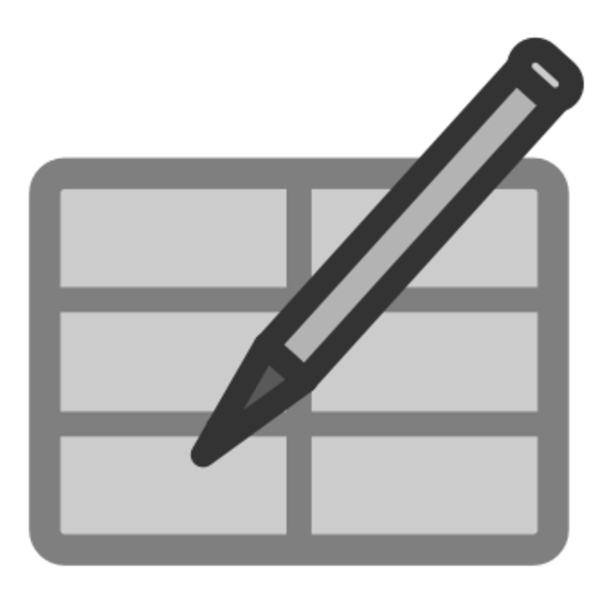


Please include in a file all important information for the sampling collection data. Information should be matching the sample codes used in the labelled sampling bags, including data of collection, site name, replicate name/number, GPS coordinates, land-use characteristics, and any other relevant observation.

Sampling Design (TBC)

Soil surface samples will be collected at 5 plots in each of the 15 sampled properties and forest areas, comprising three different land-use settings (i.e., shaded cocoa, unshaded cocoa, forest).

Plots will be established using a stratified random sampling design distributed across the sampled properties (i.e. 5 plots in each property). In each plot (each 50 × 50 m; Figure 1) four subsamples (~ 30 g per subsample, sample depth ~5 cm) will be retrieved using a trowel sterilized with a blowtorch following the recommendations provided by Taberlet et al. (2012).

These samples should then be pooled and stored in a sealed plastic container with silica gel bags for in-situ drying (and kept cool until shipping).

A total of 75 plots (corresponding to 75 samples) will be analysed, being 25 plots obtained from 5 properties for each of the land-use settings analysed.

These 75 samples will be shipped to the University of Salford, where they can be analysed by including two DNA extraction replicates per sample (for a total of 150 samples) or they can be screened for two different primer sets (150 samples analysed, being 75 samples per marker).


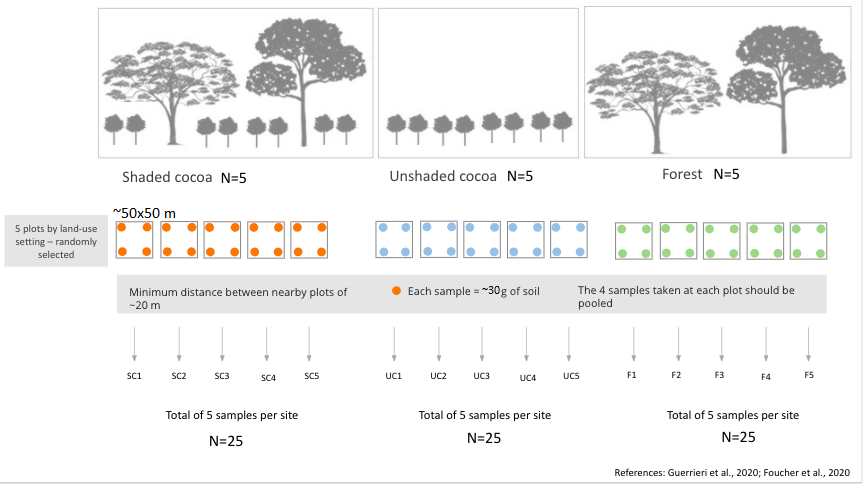

Supplement: S2 File — Detailed protocol for eDNA soil sampling. (DOCX) [file pone.0289437.s005.docx]
