# Supplementary material for: Coupling remote sensing and eDNA to monitor environmental impact: A pilot to quantify the environmental benefits of sustainable agriculture in the Brazilian Amazon
Source: PLoS One. 2024 Feb 14;19(2):e0289437. doi: 10.1371/journal.pone.0289437 (PMC10866516; doi:10.1371/journal.pone.0289437)
Supplement: S3 File — Indicator results from the vertebrate primers. (DOCX) [file pone.0289437.s006.docx]

# VERTEBRATE RESULTS

## Biodiversity Indicator 1: Number of key species due to intervention.

A small number of threatened species were identified. *Tayassu pecari* (Link), the vulnerable white-lipped peccary, was found only in cocoa fields, and *Alouatta* sp. (Lacepede), a species of decreasing howler monkeys, was found only in our forest site (IUCN, 2021). No indicator species were identified due to the low amount of data.

## Biodiversity Indicator 2: Change in abundance of keystone/ priority species due to interventions.

Only one shaded cocoa field had key species present. We found that there was no significant difference in key species abundance between cocoa fields (intervention) and pasture (counterfactuals; Pr(>Chisq) = 0.36; Fig. S1).


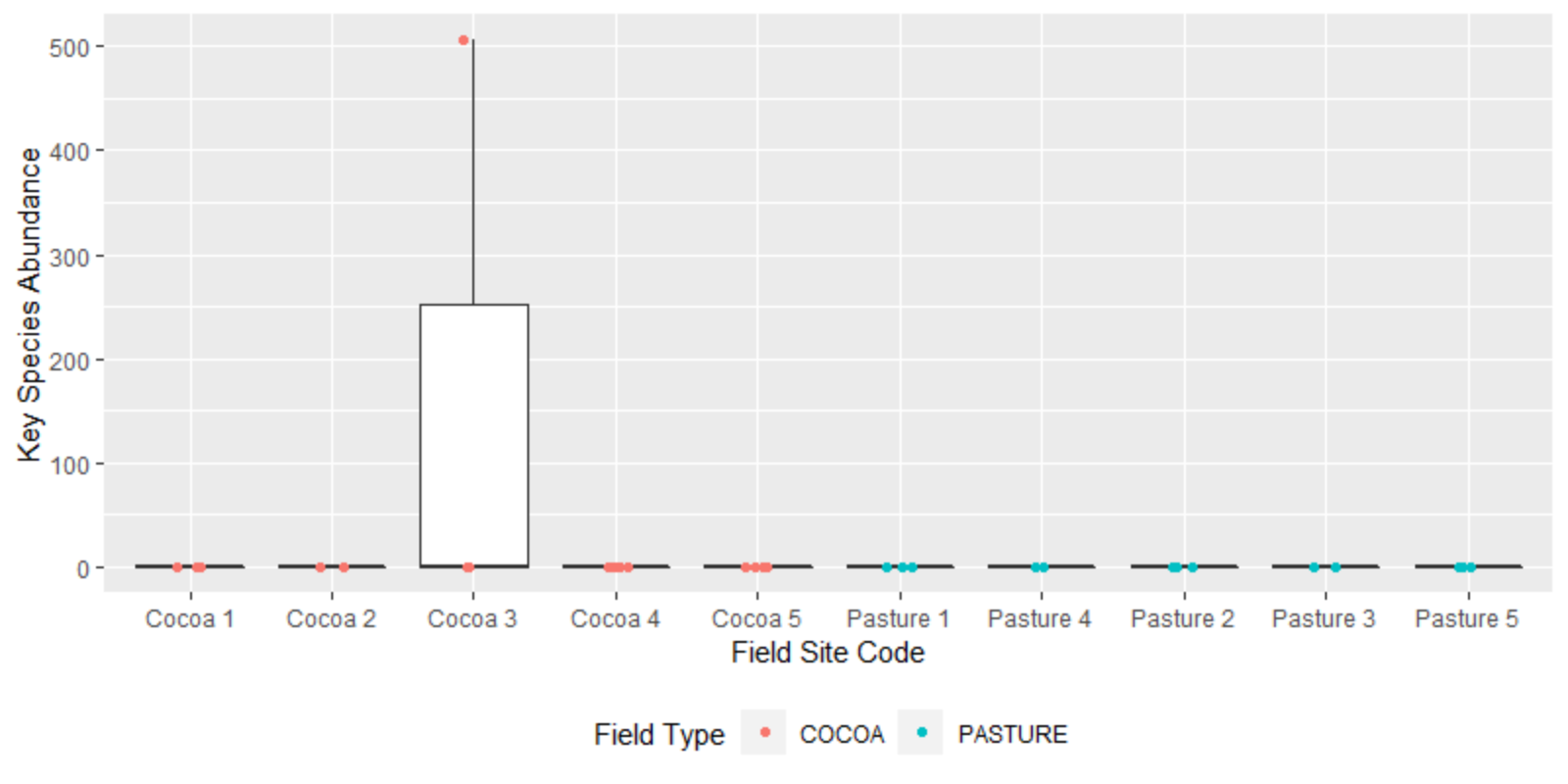


*Figure S1: Key species abundance (reads) by field site. Relative abundance is measured using the number of reads.*

## Biodiversity Indicator 3: Change in species richness due to interventions.

In addition to examining key species, or species of particular importance, we also examined the community composition of mammal populations found on cocoa fields (intervention) and pastures (counterfactual). Due to the very low number of native species detected, species accumulation curves could not be calculated and we did not capture the full species richness of the sampled locations.

The mean species richness in cocoa fields was 0.18 species (SD = 0.39), while on pastures mean species richness was 0.2 (SD = 0.41). When comparing total species richness between cocoa fields (intervention) and pasture (counterfactuals), we found that there was no significant difference (Pr(>Chisq) =0.86; Fig. S2).


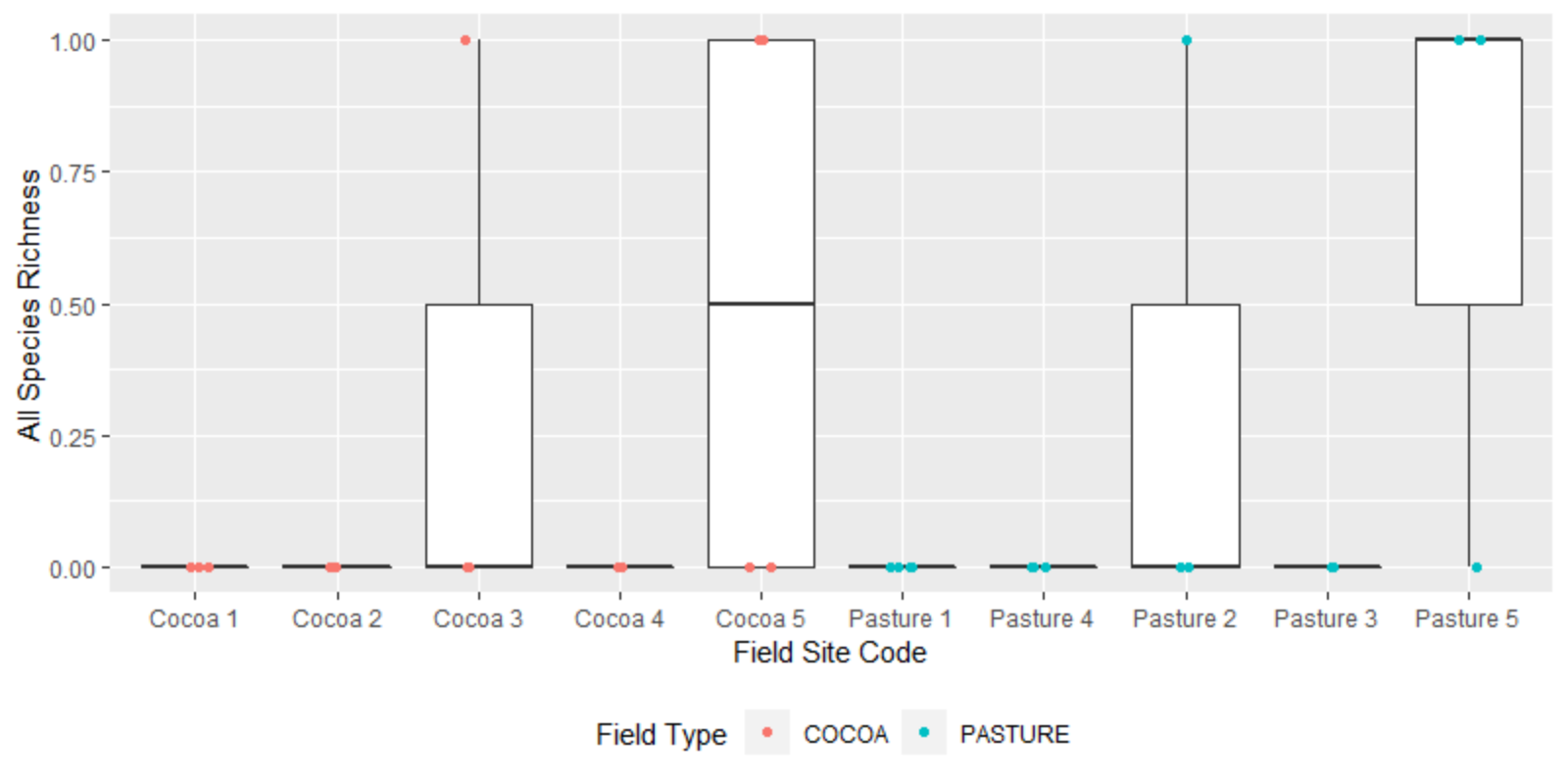


*Figure S2: Total species richness by field site.*

## Biodiversity Indicator 4: Change in biodiversity indices due to interventions.

We could not compare diversity indices between cocoa fields (intervention) and pastures (counterfactuals) due to the very low number of native species detected; only one forest site had more than one species detected.

## Biodiversity Indicator 5: Alpha diversity

We could not compare diversity indices between cocoa fields (intervention) and pastures (counterfactuals) due to the very low number of native species detected.

## Biodiversity Indicator 6: Beta diversity

Beta diversity (community dissimilarity) was based on Aitchison distances. Pairwise distances between all sampling sites (shaded cocoa, pasture, and forest) ranged from 0.80 to 9.65, where 0 represents no dissimilarity and larger distances indicate increasing dissimilarity (Fig. S3). No clear patterns in Aitchison distance were observed (Fig. S4).


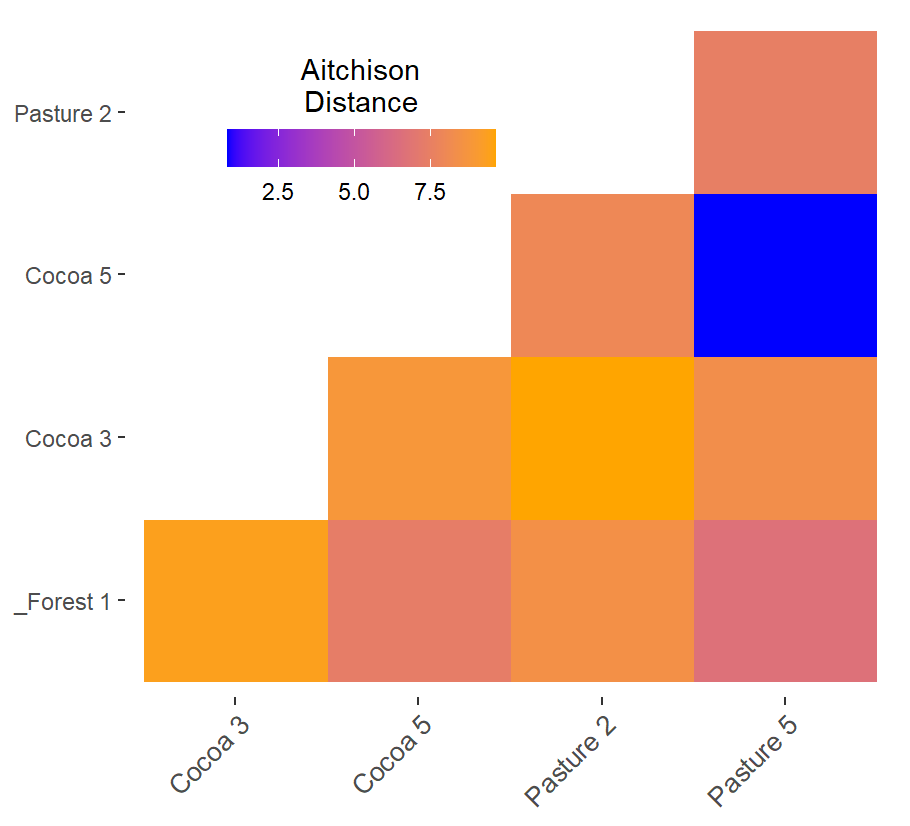


*Figure S3: Beta diversity (Aitchison distance) between each site.*


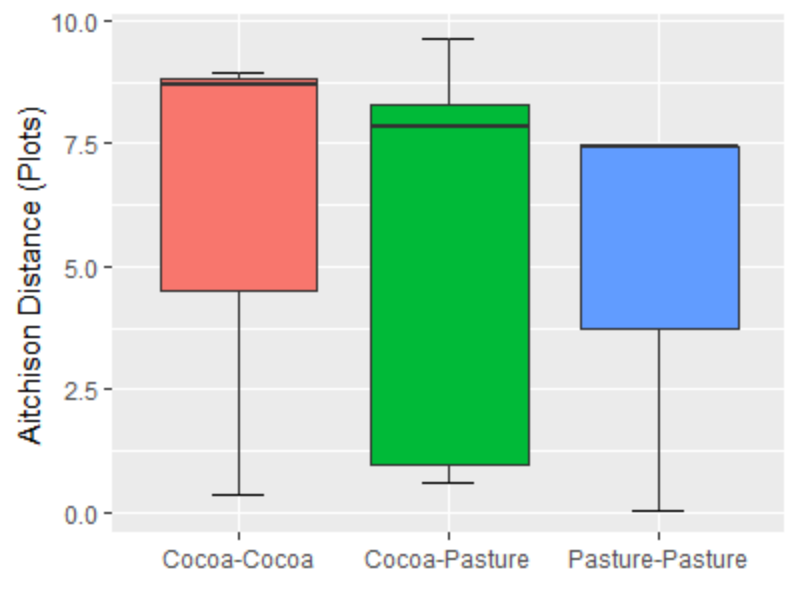


*Figure S4: Aitchison distances within treatments and between treatments.*

## Biodiversity Indicator 7: Change in beta diversity due to the intervention.

The Aitchison distance observed between shaded cocoa (intervention) and pasture (BAU) sites (3.25) was lower than the distance observed between both cocoa and forest (control) sites and pasture and forest sites (6.93 and 6.39, respectively). These differences were not significant.

## Biodiversity Indicator 8: Qualitative assessment of change in biodiversity due to the intervention

Too little data was returned to create interpretable vertebrate PCA plots.
